# Supplementary material for: Characterization of Feline Basophils on the Sysmex XN-1000V and Evaluation of a New WDF Gating Profile
Source: Animals (Basel). 2024 Nov 22;14(23):3362. doi: 10.3390/ani14233362 (PMC11639760; doi:10.3390/ani14233362)
Supplement: Supplementary file 1 [file animals-14-03362-s001.zip › Table S1.pdf]

Supplementary Table S1

Gate information of the new WDF gating strategy on the WDF channel from the Sysmex XN-1000V on the manual analysis (extended), with inclusion of the new basophil WDF gate. Abbreviations: Basoph, basophils; Eos, eosinophils; Lymph, lymphocytes; Neut, neutrophils, Mono, monoctes; TC, total cells.

TC		Mono		Neut		Eos		Lymph		Baso	
x	y	x	y	x	y	x	y	x	y	x	y
28	32	85	111	80	25	146	31	59	28	135	54
62	28	122	94	86	21	154	34	74	26	150	68
73	27	134	90	88	19	169	46	82	25	167	87
81	25	148	121	96	15	194	73	80	27	182	105
88	19	150	133	104	16	216	100	89	34	192	123
98	15	159	153	120	17	226	119	93	39	197	135
108	15	165	184	127	21	233	132	102	46	202	149
124	18	168	223	140	26	235	147	106	51	203	205
154	33	169	254	146	30	222	149	112	57	203	247
178	53	122	254	133	57	202	148	122	95	181	246
214	95	114	205	132	78	190	120	89	108	173	245
243	149	96	146	134	91	182	106	74	107	169	237
246	239	91	127	121	94	173	94	61	83	169	221
247	255			120	88	160	78	49	48	167	197
14	255			114	65	150	67	51	31	164	173
14	38			108	53	140	59			154	144

				104	51	135	53			142	109
				98	41	132	52			138	99
				89	36	145	33			134	91
				83	28					131	79
