# Supplementary figures and images for: Characterization of Feline Basophils on the Sysmex XN-1000V and Evaluation of a New WDF Gating Profile
Source: Animals (Basel). 2024 Nov 22;14(23):3362. doi: 10.3390/ani14233362 (PMC11639760; doi:10.3390/ani14233362)

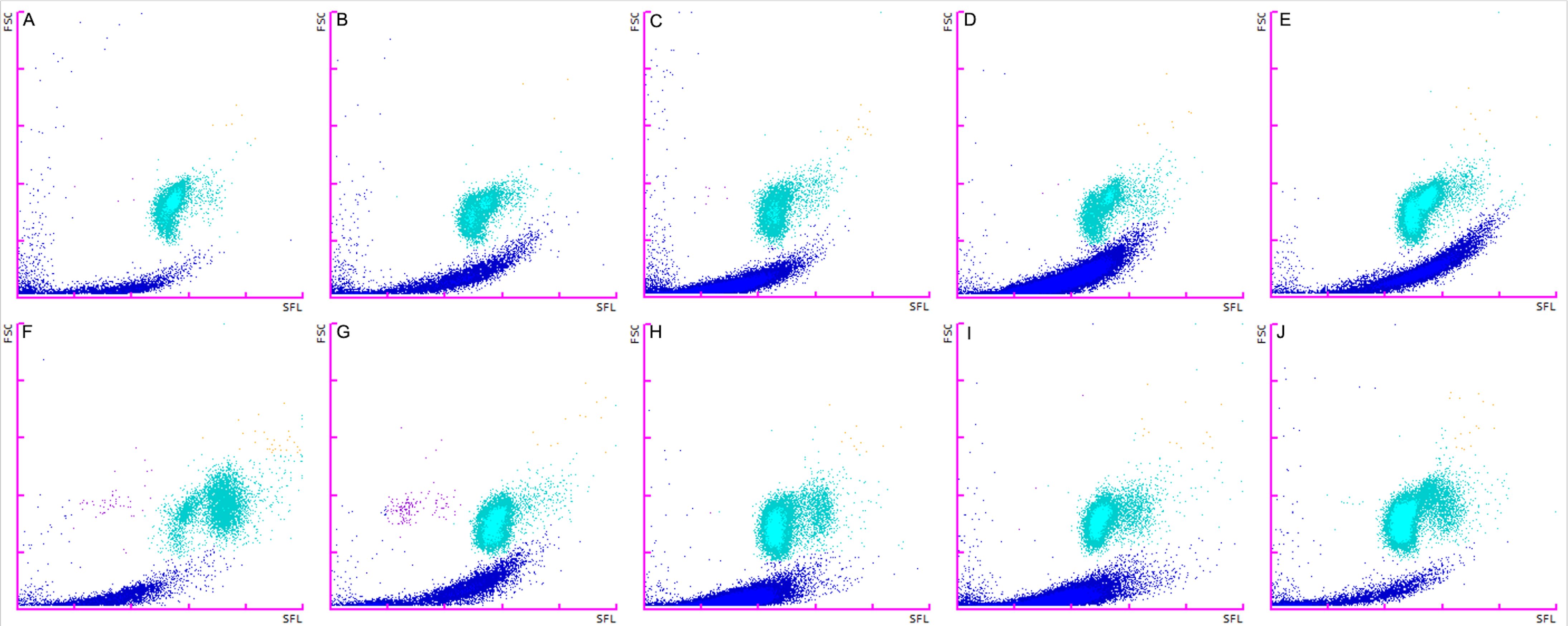

Supplement: Supplementary file 1 [file animals-14-03362-s001.zip › Figure S1.tiff]
